# Supplementary material for: Seasonal specialization drives divergent population dynamics in two closely related butterflies
Source: Nat Commun. 2023 Jun 20;14:3663. doi: 10.1038/s41467-023-39359-8 (PMC10281946; doi:10.1038/s41467-023-39359-8)
Supplement: Supplementary file 1 — Supporting information [file 41467_2023_39359_MOESM1_ESM.pdf]

Supplementary information

Seasonal specialization drives divergent population dynamics in two closely related butterflies

Loke von Schmalensee[1,2], Pauline Caillault[1], Katrín Hulda Gunnarsdóttir[1], Karl Gotthard[1,2], Philipp Lehmann[1,2,3]

[1]Department of Zoology, Stockholm University, SE-106 91, Stockholm, Sweden

[2]Bolin Centre for Climate Research, Stockholm University, SE-106 91, Stockholm, Sweden

[3]Department of Animal Physiology, Zoological Institute and Museum, University of Greifswald, D-17489, Greifswald, Germany

Contents

Appendix S1: Modelling details

- Nonlinear TPC models (p. 2)
- Priors on TPC parameters (p. 2)
- Model uncertainty (p. 2)
- Predicted microclimate-related differences in growth and development rates (pp. 2–3)

Figures S1–S9 (pp. 4–12)

References (pp. 13–14)

Appendix S1: Modelling details

Nonlinear TPC models

Researchers have long recognized that the effects of temperature on insect growth and development rates often follow nonlinear unimodal and left-skewed reaction norms[1,2]. Since then, these so-called thermal performance curves (TPCs) have been extensively studied, and the generality of their shapes has been corroborated by both theory and vast amounts of empirical data[3,4,5]. A multitude of mathematical models that describe nonlinear insect TPCs have been developed, and they must balance empirical performance (how well they fit to empirical data) with mechanistic meaning and ease of fitting[6]. Bayesian methods using Markov chain Monte Carlo (MCMC) sampling can be powerful tools for fitting such models, especially in combination with informative priors, but it has so far been under-utilized in the published literature. The Lobry-Rosso-Flandrois model used here have the particular benefit that all four of its parameters (T_{min}, T_{opt}, T_{max} and R_{opt}) are biologically meaningful and easily interpretable, simplifying incorporation of prior knowledge[7,8].

Priors on TPC parameters

In many nonlinear models, informative priors for parameter values are necessary to deal with identifiability issues and model convergence. Luckily, in the field of insect thermal biology, there are often vast amounts of prior knowledge to be gathered[5,9,10,11,12] and models with interpretable parameters (e.g. T_{min}, T_{opt}, and T_{max}) allows for expressing this knowledge through prior specifications on familiar scales. For example, if a researcher believes with great confidence that T_{min} for development must be lower than 20°C – perhaps because they have previously reared multiple cohorts at that temperature – it is appropriate to include that information in the prior distribution of the T_{min} parameter. Furthermore, priors can be used to avoid exploring nonsensical parameter combinations, like $T_{min} > T_{max}$. It is important to keep in mind that traditional methods of fitting nonlinear thermal performance curves, e.g. through optimizers minimizing squared residuals, are not free from these limitations. On the contrary, they can be highly sensitive to initial parameter values, which in turn might need to be visually estimated from the data itself[5], and small differences in starting values could lead to model convergence at different local optima. For the specific prior distributions used here, see Supplementary Data 1.

Model uncertainty

It is well-known that estimates of T_{max} for performance-traits are associated with relatively high levels of uncertainty due to high mortality rates at those temperatures[13]. Therefore, differences in T_{max} between species, populations or life stages are difficult to assess using conventional significance-level tests, and when T_{max} is estimated using empirical nonlinear TPC models, its uncertainty is reported in various ways, and often not at all (see Rebaudo *et al.*[5] for a comprehensive reference list). Indeed, major comparative studies and reviews have, understandably, focused on the left part of the TPC, likely since T_{min} can easily be approximated using a linear function[12,14]. Instead, we here use a methodology that allows for appropriate representation of model uncertainty (including consideration of group-level variation) – something which thus far has been lacking from the insect TPC literature – and perform multiple independent experiments and analyses.

Predicted microclimate-related differences in growth and development rates

To test whether differences in oviposition preference between *Pieris rapae* and *P. napi* corresponded to differences in the relative performance of their offspring, we predicted habitat-specific average performance for larvae of both species during the month following oviposition. We first calculated hourly

larval development and growth rates for *P. rapae* and *P. napi* in each microhabitat, through rate summation[15] using hourly microclimate measurements recorded 31 days after the end of the oviposition experiment. We then calculated the average (over the 31 days) hourly development and growth rates for *P. rapae* and *P. napi* in each microhabitat. Last, we divided the average development and growth rates of *P. rapae* with those of *P. napi* in each microhabitat, resulting in a ratio with higher values (> 1) representing larger proportional performance differences in *P. rapae's* favor. We then overlaid the distribution of the *P. rapae* / *P. napi* development and growth rate ratios with the estimated oviposition preference (Fig. 2 in the main manuscript). The ratios increased with oviposition temperature for both development and growth rates (Fig. S8). This suggests that *P. rapae* has a larger competitive edge over *P. napi* in warmer microhabitats than in colder microhabitats. Under this (reasonable) assumption – that rapid larval development and growth are favorable when competing for food on a common host plant – selection should, from an optimality perspective, favor divergent oviposition preferences in the two species, with *P. rapae* preferring warmer microclimates than do *P. napi* (as seen in the data; Fig. S8).

Figures S1–S9

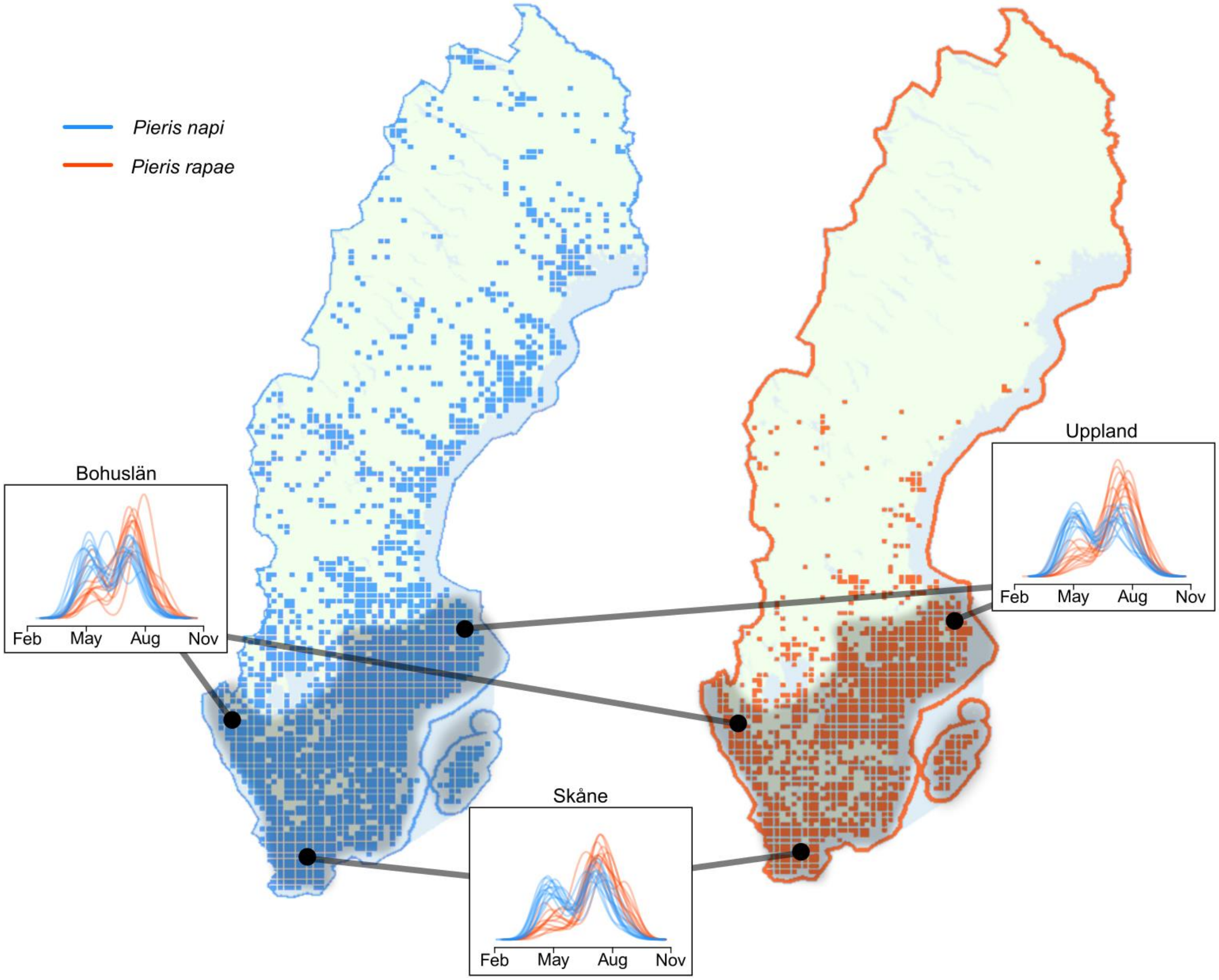

Figure S1. Figure showing the geographical locations of *Pieris rapae* (orange) and *P. napi* (blue) observations in Sweden between years 2010–2020[16]. Raster map adapted from www.artportalen.se. Shaded areas show the geographical regions from where citizen science were included, representing the common range of the two species. The provinces included in the analyses were Uppland, Västmanland, Södermanland, Närke, Östergötland, Västergötland, Bohuslän, Småland, Halland, Blekinge, Skåne, Öland, and Gotland. Density plots are shown to demonstrate that the difference in population dynamics between the two species persists throughout their common range.

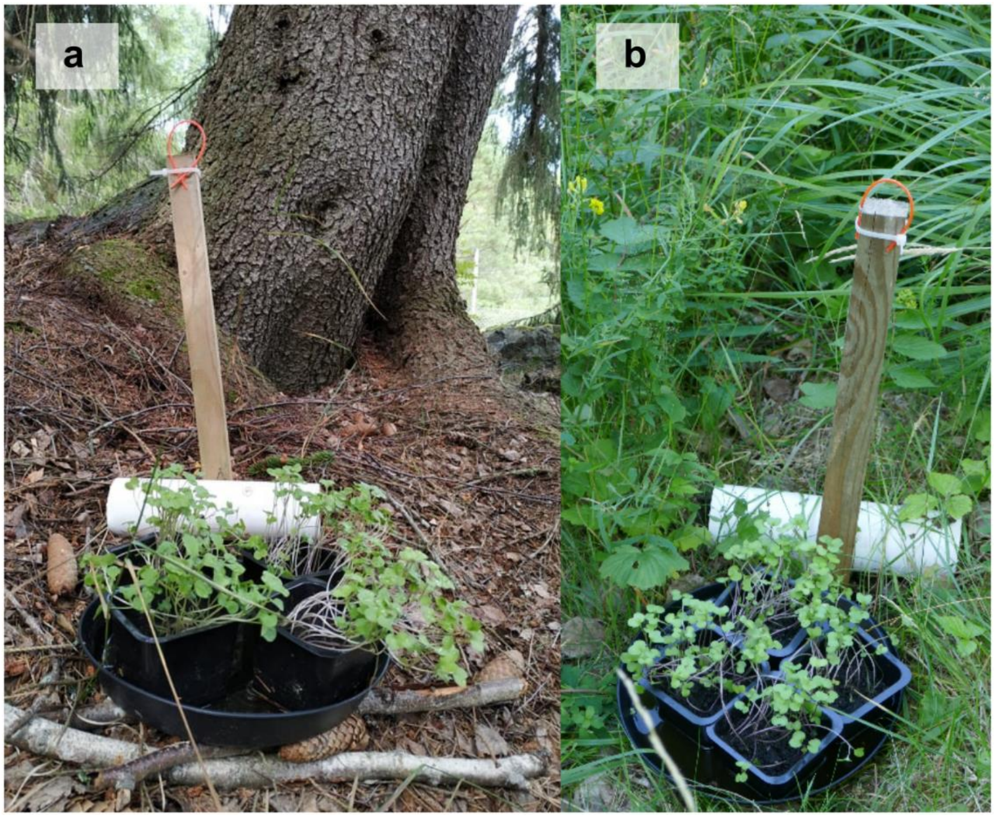

Figure S2. Pictures showing the setup of the field oviposition experiment at **a** a sheltered forest site and **b** a semi-exposed site. Temperature loggers (EL USB-1) were placed in the white PVC tubes next to the transplanted host plants (*Brassica napus napus*).

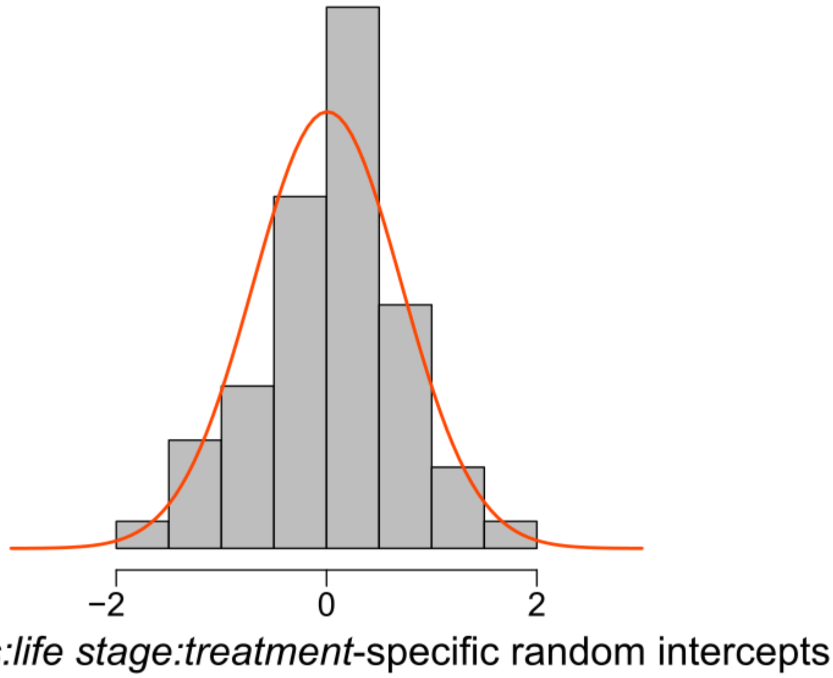

Figure S3. The histogram shows the distribution of random intercepts at for each unique combination of species, life stage, and temperature treatment, and the red curve represents the probability density function of a normal distribution with the same mean and variance as the data in the histogram. The concordance between the two indicate that the modelling approach is appropriate, and that randomly distributed batch effects dominate the "fixed" effects caused by life stage-differences.

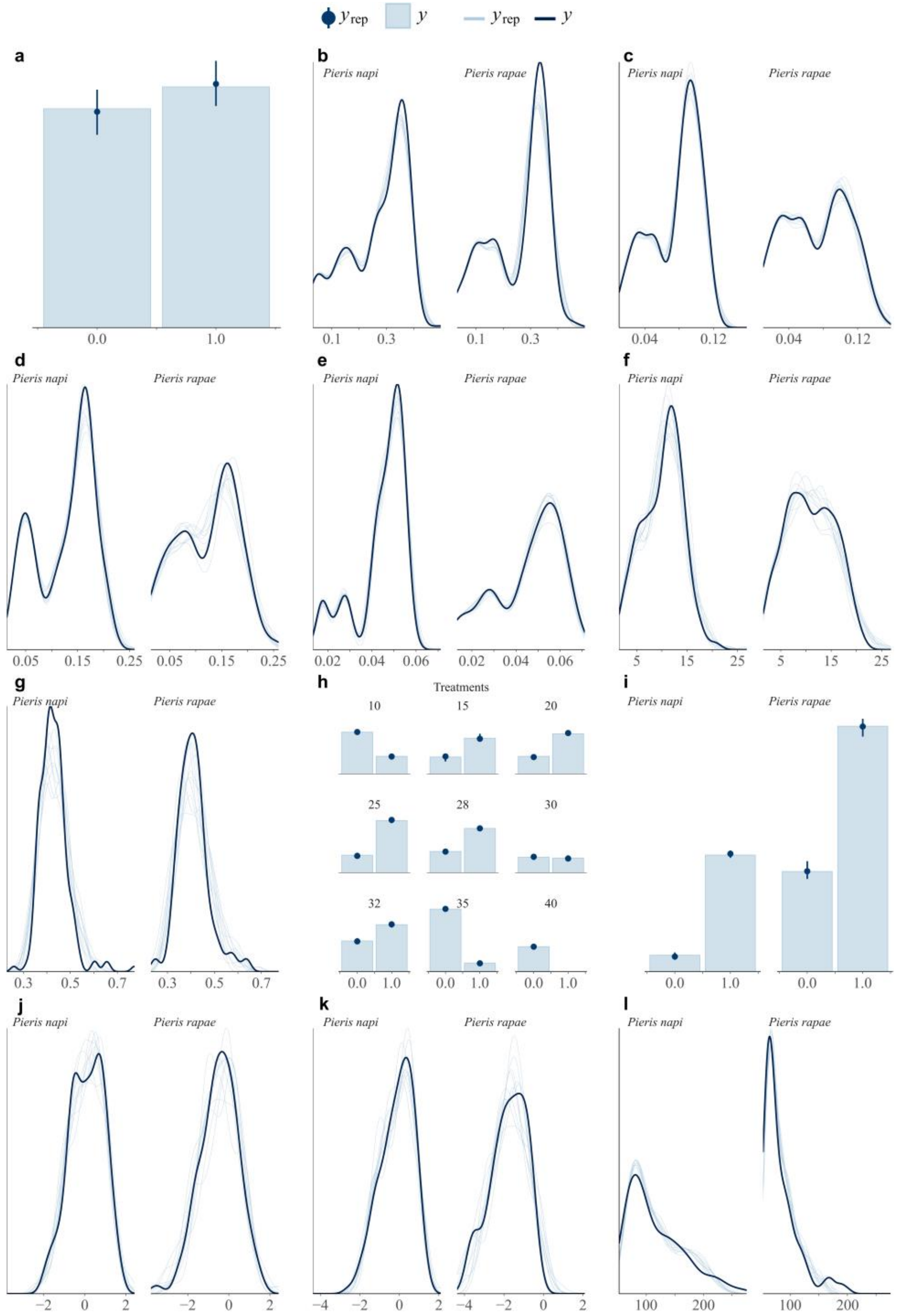

Figure S4. Posterior predictive checks for the **a** oviposition model, **b** egg development rate model, **c** larval development rate model, **d** pupal development rate model, **e** ontogenetic development rate model, **f** larval growth rate model, **g** pupal mass retention model, **h** developmental survival model, **i** overwintering model, **j/k** summer and winter citizen science-data models, and **l** the citizen science observations over time model. Response variable values are plotted on the x-axes and their frequencies are plotted on the y-axes. In bar plots, blue bars represent counts of the real data, and black dots with error bars represent distributions (medians and range) of data simulated from the models ($n_{simulations}$ = 10). In line plots, thick black lines represent distributions of the real data, and thin blue lines represent distributions of data simulated from the model ($n_{simulations}$ = 10).

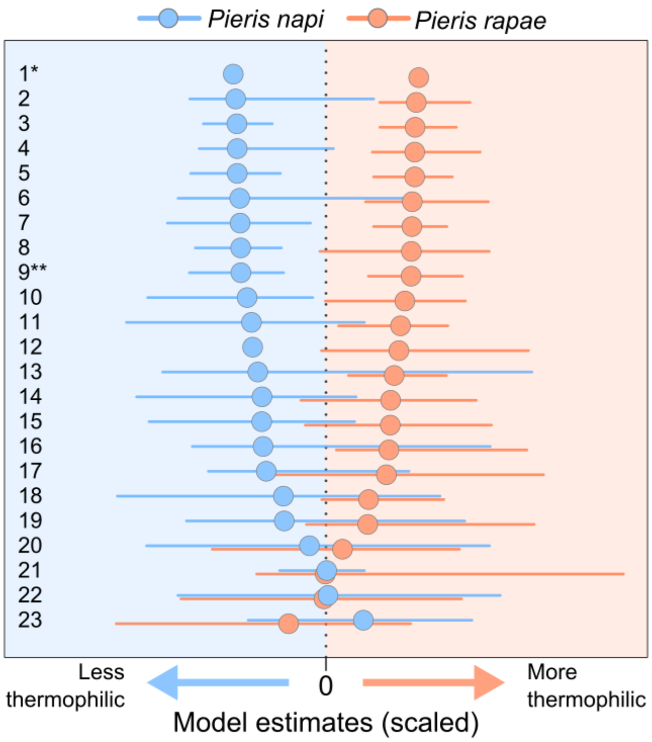

Figure S5. Parameter point estimates (posterior modes, points) and 90% credible intervals (lines). The point estimates have been centered on 0, and the variance has been scaled (dived by the total standard deviation of the posterior samples for a given parameter). The sign of the parameter has been adjusted so that a right shift on the x-axis corresponds to a more thermophilic trait value (e.g. higher T_{max}, lower overwintering survival, or higher summer population growth per capita). Following numbers declare which parameter corresponds to which data. (1) Intercept, survival development at 35°C (n = 255 *Pieris rapae*, n = 192 *P. napi*); (2) T_{opt}, pupal development rate (n = 133 *P. rapae*, n = 280 *P. napi*); (3) Intercept, winter population decline per capita (n = 50 *P. rapae*, n = 127 *P. napi*); (4) T_{opt}, larval development rate (n = 288 *P. rapae*, n = 236 *P. napi*); (5) T_{min}, ontogenetic development rate (n = 98 *P. rapae*, n = 172 *P. napi*); (6) T_{max}, pupal development rate (n = 133 *P. rapae*, n = 280 *P. napi*); (7) T_{min}, pupal development rate (n = 133 *P. rapae*, n = 280 *P. napi*); (8) Intercept, summer population growth per capita (n = 92 *P. rapae*, n = 147 *P. napi*); (9) Temperature slope, oviposition preference (n = 121 *P. rapae*, n = 110 *P. napi*); (10) Intercept, survival pupal overwintering (n = 652 *P. rapae*, n = 251 *P. napi*); (11) T_{min}, egg development rate (n = 258 *P. rapae*, n = 311 *P. napi*); (12) T_{max}, ontogenetic development rate (n = 98 *P. rapae*, n = 172 *P. napi*); (13) T_{opt}, egg development rate (n = 258 *P. rapae*, n = 311 *P. napi*); (14) Intercept, survival development at 32°C (n = 191 *P. rapae*, n = 297 *P. napi*); (15) temperature slope, pupal mass retention (n = 125 *P. rapae*, n = 279 *P. napi*); (16) T_{max}, larval development rate (n = 288 *P. rapae*, n = 236 *P. napi*); (17) T_{opt}, larval growth rate (n = 283 *P. rapae*, n = 234 *P. napi*); (18) T_{max}, egg development rate (n = 258 *P. rapae*, n = 311 *P. napi*); (19) T_{max}, larval growth rate (n = 283 *P. rapae*, n = 234 *P. napi*); (20) T_{min}, larval development rate (n = 288 *P. rapae*, n = 236 *P. napi*); (21) T_{opt}, ontogenetic development rate (n = 98 *P. rapae*, n = 172 *P. napi*); (22) Intercept, survival development at 10°C (n = 170 *P. rapae*, n = 204 *P. napi*); (23) T_{min}, larval growth rate (n = 283 *P. rapae*, n = 234 *P. napi*).

* No survival in *Pieris napi*.
** Slope in oviposition experiment indicating with high confidence that increasing temperatures lead to increasing probabilities of eggs being *P. rapae* (instead of *P. napi*; Fig. 1). For visualization purposes, the opposite slope (showing that *P. napi* prefer colder temperatures), has also been plotted.

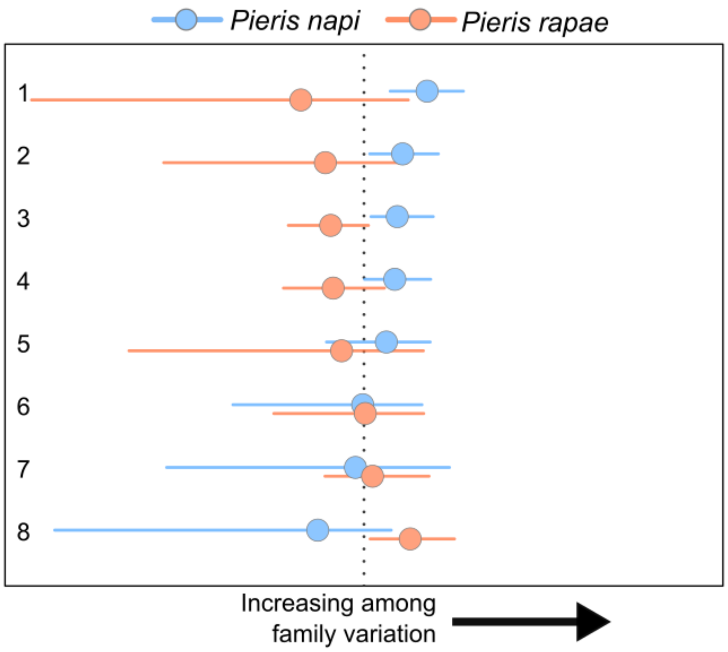

Figure S6. Estimated among-family variation (logarithmized standard deviations) for *Pieris napi* and *P. rapae* traits. Points represent point estimates (posterior modes) and lines represent 90% credible intervals. Following numbers declare which trait corresponds to which data. (1) Pupal development rate (n = 133 *Pieris rapae*, n = 280 *P. napi*); (2) ontogenetic development rate (n = 98 *P. rapae*, n = 172 *P. napi*); (3) egg development rate (n = 258 *P. rapae*, n = 311 *P. napi*); (4) survival during development (n = 1536 *P. rapae*, n = 1669 *P. napi*); (5) pupal mass retention (n = 125 *P. rapae*, n = 279 *P. napi*); (6) larval development rate (n = 288 *P. rapae*, n = 236 *P. napi*); (7) survival during overwintering (n = 652 *P. rapae*, n = 251 *P. napi*); (8) larval growth rate (n = 283 *P. rapae*, n = 234 *P. napi*).

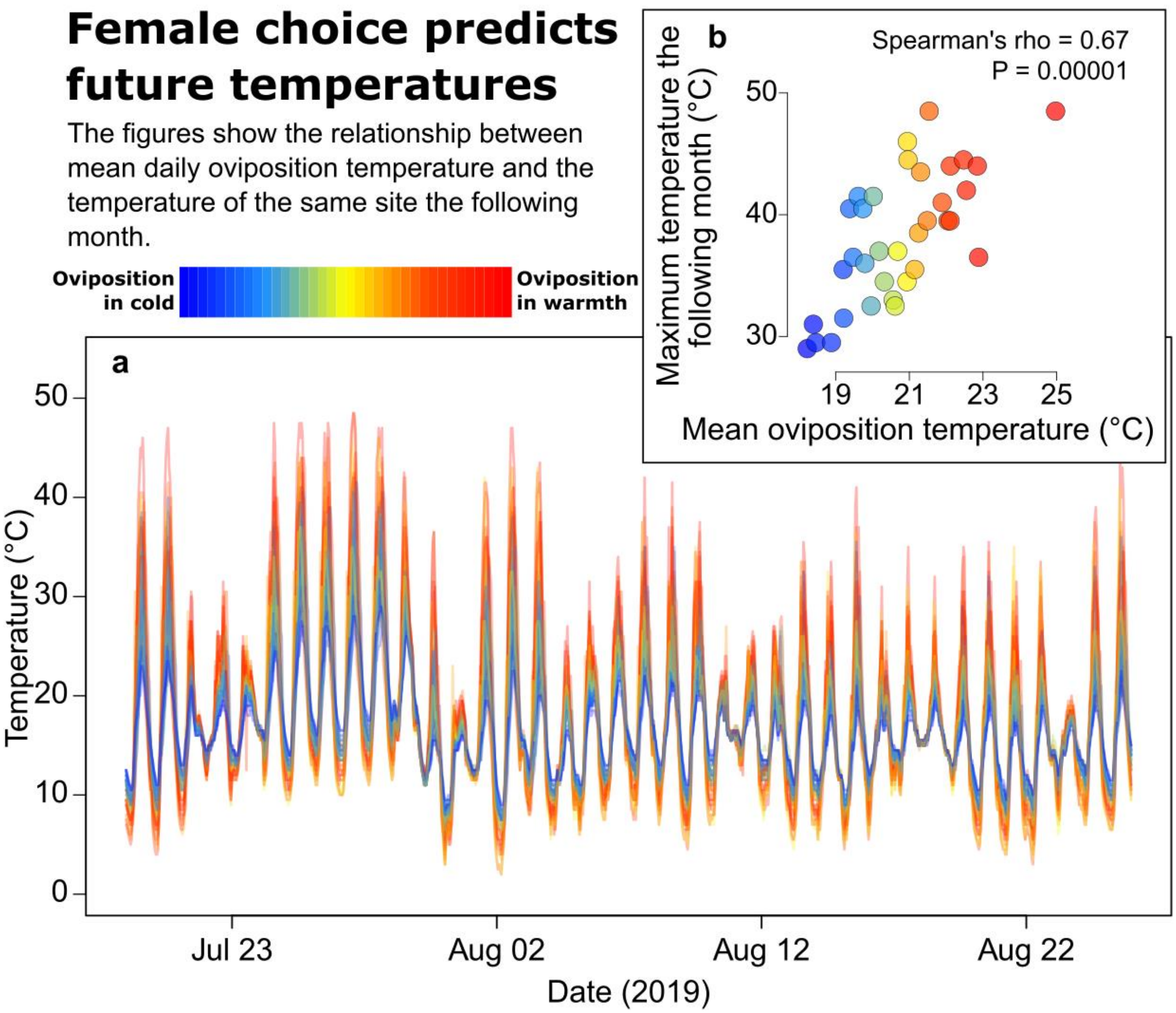

Figure S7. Demonstration of how *Pieris rapae* and *P. napi* oviposition microhabitat choice is related to the future microclimatic temperatures experienced by the offspring. **a** How microclimate varies in each oviposition microhabitat one month following the end of the oviposition experiment. Each thermal regime is colored by the mean daily temperature at that microhabitat during oviposition (blue represents oviposition in cold temperatures, red represents oviposition in warm temperatures). When eggs are laid in relatively warm conditions, as preferred by *P. rapae*, the microclimate experienced by the offspring tends to be highly variable with frequent fluctuations into extreme warm temperatures. When eggs are laid in relatively cold conditions, as preferred by *P. napi*, the microclimates experienced by the offspring tend to have low temperature variance and mean temperatures. **b** How oviposition temperature relates to maximum microclimate temperature in the same microhabitat the following month. Oviposition in warm microhabitats result in the offspring experiencing high extreme temperatures, and vice versa (Spearman's rank correlation, two-sided P = 0.00001). Colors in **b** correspond to those in **a**. Temperature data have been previously published[17].

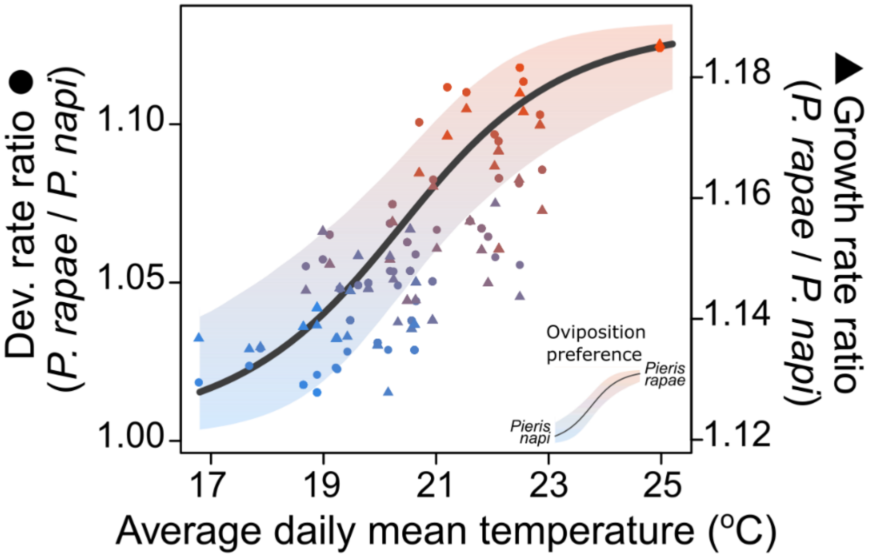

Figure S8. The relationship between oviposition preference (logistic curve with 90% credible interval band, corresponding to Fig. 2 in the main manuscript), and ratios of predicted *Pieris rapae* to *P. napi* larval development rates (circles, left y-axis) and growth rates (triangles, right y-axis) for each microhabitat and oviposition period. Mean oviposition temperature is denoted on the x-axis. Since all ratios are > 1, higher ratios represent larger proportional performance differences in favor of *P. rapae*. Temperature data have been previously published[17].

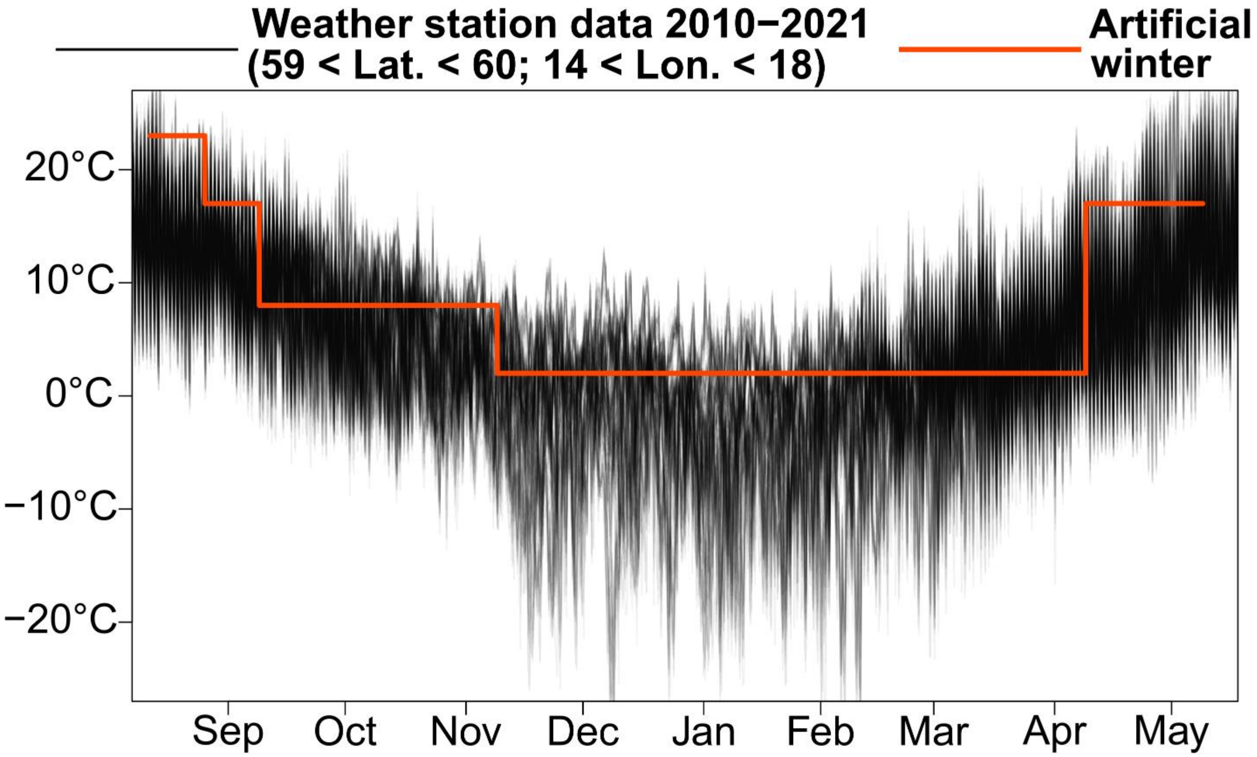

Figure S9. The artificial thermal regime (red line) used for assessing *Pieris rapae* and *P. napi* overwintering success overlaid on 11 years of winter temperature data from Swedish weather stations (from the same region as the butterfly populations, 59 < Latitude < 60, 14 < Longitude < 18; climate data from SMHI[18], n = 20). Note that the artificial winter regime is shifted in time to show its match with natural winter conditions.

References

1. Cook, W. C. Some Effects of Alternating Temperatures on the Growth and Metabolism of Cutworm Larvae. *J. Econ. Entomol.* **20**, 769–782 (1927).

2. Janisch, E. The influence of temperature on the life-history of insects. *Ecol. Entomol.* **80**, 137–168 (1932).

3. Huey, R.B. & Kingsolver, J.G. Evolution of Thermal Sensitivity of Ectotherm Performance. *Trends Ecol. Evol.* **4**, 131–135 (1989).

4. Logan, J. A., Wollkind, D. J., Hoyt, S. C. & Tanigoshi, L. K. An Analytic Model for Description of Temperature Dependent Rate Phenomena in Arthropods. *Environ. Entomol.* **5**, 1133–1140 (1976).

5. Rebaudo, F., Struelens, Q. & Dangles, O. Modelling temperature-dependent development rate and phenology in arthropods: The devRate package for r. *Methods Ecol. Evol.* **9**, 1144–1150 (2018).

6. Rebaudo, F. & Rabhi, V.B. Modeling temperature-dependent development rate and phenology in insects: review of major developments, challenges, and future directions. *Entomol. Exp. Appl.* **166**, 607–617 (2018).

7. Ratkowsky, D.A. & Reddy G V.P. Empirical model with excellent statistical properties for describing temperature-dependent developmental rates of insects and mites. *Ann. Entomol. Soc. Am.* **110**, 302–309 (2017).

8. Rosso, L., Lobry, J. R. & Flandrois, J. P. An unexpected correlation between cardinal temperatures of microbial growth highlighted by a new model. *J. Theor. Biol.* **162**, 447–463 (1993).

9. Addo-Bediako, A., Chown, S.L. & Gaston, K.J. Thermal tolerance, climatic variability and latitude. *Proc. Royal Soc. B* **267**, 739–745 (2000).

10. Chown, S.L., Addo-Bediako, A. & Gaston, K.J. Physiological variation in insects: Large-scale patterns and their implications. *Comp. Biochem. Physiol. B, Biochem. Mol. Biol.* **131**, 587–602 (2002).

11. Hoffmann, A.A., Chown, S.L. & Clusella-Trullas, S. Upper thermal limits in terrestrial ectotherms: How constrained are they? *Funct. Ecol.* **27**, 934–949 (2013).

12. Jarošík, V., Honěk, A., Magarey, R. D. & Skuhrovec, J. Developmental database for phenology models: Related insect and mite species have similar thermal requirements. *J. Econ. Entomol.* **104**, 1870–1876 (2011).

13. Régnière, J., Powell, J., Bentz, B. & Nealis, V. Effects of temperature on development , survival and reproduction of insects: Experimental design, data analysis and modeling. *J. Insect Physiol.* **58**, 634–647 (2012).

14. Buckley, L. B., Arakaki, A. J., Cannistra, A. F., Kharouba, H. M. & Kingsolver, J. G. Insect Development, Thermal Plasticity and Fitness Implications in Changing, Seasonal Environments. *Integr. Comp. Biol.* **57**, 988–998 (2017).

15. von Schmalensee, L., Hulda Gunnarsdóttir, K., Näslund, J., Gotthard, K. & Lehmann, P. Thermal performance under constant temperatures can accurately predict insect development times across naturally variable microclimates. *Ecol. Lett.* **24**, 1633–1645 (2021).

16. ArtDatabanken. *Artportalen (Species Observation System)*. https://www.artportalen.se (2022).

17. Greiser, C., von Schmalensee, L., Lindestad, O., Gotthard, K. & Lehmann, P. Microclimatic variation affects developmental phenology, synchrony and voltinism in an insect population. *Funct. Ecol.* **36**, 3036–3048 (2022).

18. SMHI. *Swedish Meteorological and Hydrological Institute*. http://www.smhi.se (2023).
