# Supplementary material for: Seasonal specialization drives divergent population dynamics in two closely related butterflies
Source: Nat Commun. 2023 Jun 20;14:3663. doi: 10.1038/s41467-023-39359-8 (PMC10281946; doi:10.1038/s41467-023-39359-8)
Supplement: Supplementary file 3 — Description of additional supplementary files [file 41467_2023_39359_MOESM3_ESM.pdf]

# <u>Description of additional supplementary files</u>

File Name: Supplementary Data 1
Description: Table of point estimates (posterior modes), 90% credible intervals, and priors for all parameters in all models.
