# Supplementary material for: Seasonal specialization drives divergent population dynamics in two closely related butterflies
Source: Nat Commun. 2023 Jun 20;14:3663. doi: 10.1038/s41467-023-39359-8 (PMC10281946; doi:10.1038/s41467-023-39359-8)
Supplement: Supplementary file 5 — Reporting Summary [file 41467_2023_39359_MOESM5_ESM.pdf]

nature portfolio

Corresponding author(s): Loke von Schmalensee
Last updated by author(s): May 29, 2023

Reporting Summary

Nature Portfolio wishes to improve the reproducibility of the work that we publish. This form provides structure for consistency and transparency in reporting. For further information on Nature Portfolio policies, see our Editorial Policies and the Editorial Policy Checklist.

Statistics

For all statistical analyses, confirm that the following items are present in the figure legend, table legend, main text, or Methods section.

n/a	Confirmed	
☐	☒	The exact sample size (*n*) for each experimental group/condition, given as a discrete number and unit of measurement
☐	☒	A statement on whether measurements were taken from distinct samples or whether the same sample was measured repeatedly
☒	☐	The statistical test(s) used AND whether they are one- or two-sided<br>*Only common tests should be described solely by name; describe more complex techniques in the Methods section.*
☐	☒	A description of all covariates tested
☐	☒	A description of any assumptions or corrections, such as tests of normality and adjustment for multiple comparisons
☐	☒	A full description of the statistical parameters including central tendency (e.g. means) or other basic estimates (e.g. regression coefficient) AND variation (e.g. standard deviation) or associated estimates of uncertainty (e.g. confidence intervals)
☐	☒	For null hypothesis testing, the test statistic (e.g. *F*, *t*, *r*) with confidence intervals, effect sizes, degrees of freedom and *P* value noted<br>*Give P values as exact values whenever suitable.*
☐	☒	For Bayesian analysis, information on the choice of priors and Markov chain Monte Carlo settings
☐	☒	For hierarchical and complex designs, identification of the appropriate level for tests and full reporting of outcomes
☒	☐	Estimates of effect sizes (e.g. Cohen's *d*, Pearson's *r*), indicating how they were calculated

Our web collection on statistics for biologists contains articles on many of the points above.

Software and code

Policy information about availability of computer code

Data collection	No specific software or code was used to collect the data.
Data analysis	The probabilistic programming language Stan was used to fit bayesian models through the R (version 4.1.3) package brms (version 2.18.0). For data processing and visualization, the packages tidyverse (version 1.3.2), lubridate (version 1.8.0) and bayestestR (version 0.13.0) were used. All code used to produce the results is available in the Figshare repository [https://doi.org/10.6084/m9.figshare.22657069].

For manuscripts utilizing custom algorithms or software that are central to the research but not yet described in published literature, software must be made available to editors and reviewers. We strongly encourage code deposition in a community repository (e.g. GitHub). See the Nature Portfolio guidelines for submitting code & software for further information.

Data

Policy information about availability of data

All manuscripts must include a data availability statement. This statement should provide the following information, where applicable:

- Accession codes, unique identifiers, or web links for publicly available datasets
- A description of any restrictions on data availability
- For clinical datasets or third party data, please ensure that the statement adheres to our policy

All data is available in the Figshare repository [https://doi.org/10.6084/m9.figshare.22657069].

Human research participants

Policy information about studies involving human research participants and Sex and Gender in Research.

Reporting on sex and gender	N/A
Population characteristics	N/A
Recruitment	N/A
Ethics oversight	N/A

Note that full information on the approval of the study protocol must also be provided in the manuscript.

Field-specific reporting

Please select the one below that is the best fit for your research. If you are not sure, read the appropriate sections before making your selection.

☐ Life sciences ☐ Behavioural & social sciences ☒ Ecological, evolutionary & environmental sciences

For a reference copy of the document with all sections, see nature.com/documents/nr-reporting-summary-flat.pdf

Ecological, evolutionary & environmental sciences study design

All studies must disclose on these points even when the disclosure is negative.

Study description	We compared two butterfly species (Pieris rapae/Pieris napi, n(experiments)=2457/2041, n(citizen science)=5712/20040, respectively). Logistic/linear regressions and nonlinear models were fitted using Bayesian methods, hierarchical variance structures were used where appropriate (e.g. family variation within species).
Research sample	The research samples consisted of Pieris rapae and Pieris napi (Lepidoptera; Pieridae) individuals: eggs laid by wild females, individuals reared in the laboratory, and individuals observed in the wild by citizen scientists (ArtDatabanken, https://www.artportalen.se/). The samples were gathered to compare the two species. Laboratory and field experiments were done on populations from Stockholm, Sweden (WGS84 decimal: Lat. 59.368, Lon. 18.061).
Sampling strategy	Both Pieris rapae and Pieris napi are easy to rear in the laboratory, and sample sizes were thus mainly limited by climate cabinet space and work load. We aimed for sufficient replication of random factor levels within treatments (e.g. at least 9 containers within each combination of temperature treatment and species). In one treatment (40°C), where sample sizes were limited, the lowest sample sizes were used in the pupal stage, since they are generally less affected by confounding effects (they do not feed or move). In order to better capture nonlinearities in the temperature response, we added two high-temperature treatments to the Pieris napi data in 2019. Citizen science data from the sympatric Swedish range of the butterflies from 2010 and onward were used, because the number of reported observations has been highest the last decade.
Data collection	The citizen science data were downloaded from https://www.artportalen.se/ by LvS. Field oviposition data were collected by PC and LvS. Pieris rapae development data were collected by KHG in the laboratory. Pieris napi development data were collected by LvS in the laboratory. Diapause data were collected by LvS in the laboratory.
Timing and spatial scale	Experiments and sampling were performed 2018 and 2019. Experimental animals were collected during summer from a field near Stockholm University, Sweden (WGS84 decimal: Lat. 59.368, Lon. 18.061). Field experiments were performed during summer in a heterogeneous area in the cultural landscape of Södermanland, Sweden (WGS84 decimal: Lat. 58.973111, Lon. 17.155472; ~26 ha). Experiments were started late summer and went on until experimental animals had either completed development or died. Citizen science data were collected from observations in the sympatric Swedish range of the butterflies between 2010-2021, since the number of reported observations has been highest the last decade.
Data exclusions	Data points were excluded from specific analyses where individual traits could not be estimated. For example, development rate could not be calculated for dead individuals, and mass loss during the pupal stage could not be calculated for individuals that failed to eclose properly. Thus, these individuals were removed from the respective models. Otherwise, no data were excluded.
Reproducibility	The experiments were not reproduced. However, additional Pieris napi data from two temperatures were added in 2019, which followed the expected pattern if results are reproducible.
Randomization	We distributed individuals of genetic families evenly across temperature treatments. Within families, individuals were randomly assigned to different treatments since we could not differentiate between them. For example, the larval treatment starts with newly hatched larvae which are very small, and to our eyes impossible to distinguish from one another. Eggs were oviposited on leaves by female butterflies, and the leaves were removed when a sufficient number of eggs had been laid. Sex could not be determined before starting the experiment, but was accounted for in the modelling.

Blinding	Blinding was not used in this study both for reasons stated above, and because of the fact that measured response variables are easy to objectively quantify (e.g. development time and pupation mass) and therefore not subject to biases from the observer.

Did the study involve field work? [x] Yes [ ] No

Field work, collection and transport

Field conditions	Field conditions were generally warm and sunny when butterflies were collected, since that is when they tend to fly. During the field experiment, weather was variable, as it was carried out for several days.
Location	Sampling for laboratory experiments was done in Stockholm, Sweden (WGS84 decimal: Lat. 59.368, Lon. 18.061). The field experiment was performed in the cultural landscape of Södermanland, Sweden (WGS84 decimal: Lat. 58.973111, Lon. 17.155472)
Access & import/export	Neither of the two butterfly species (Pieris rapae and Pieris napi) are protected, and require no permissions to catch in Sweden. The area for the field experiment is owned by the institution where this work was done, Stockholm University.
Disturbance	Both of the two species are abundant in the wild, and the field experiment involved rapeseed, a plant that is commonly cultivated in the study area. We estimate that our studies have had a minimal disturbance on the surroundings.

Reporting for specific materials, systems and methods

We require information from authors about some types of materials, experimental systems and methods used in many studies. Here, indicate whether each material, system or method listed is relevant to your study. If you are not sure if a list item applies to your research, read the appropriate section before selecting a response.

Materials & experimental systems

n/a	Involved in the study
[x]	[ ] Antibodies
[x]	[ ] Eukaryotic cell lines
[x]	[ ] Palaeontology and archaeology
[ ]	[x] Animals and other organisms
[x]	[ ] Clinical data
[x]	[ ] Dual use research of concern

Methods

n/a	Involved in the study
[x]	[ ] ChIP-seq
[x]	[ ] Flow cytometry
[x]	[ ] MRI-based neuroimaging

Animals and other research organisms

Policy information about studies involving animals; ARRIVE guidelines recommended for reporting animal research, and Sex and Gender in Research

Laboratory animals	Offspring of field collected butterflies (Pieris napi and Pieris rapae) were reared for a maximum of two additional generations in the lab. Long-days (22L: 2D) with intermediate temperature (23°C) were used as ambient temperatures for rearings. For the different temperature treatments, temperature varied (10, 15, 20, 25, 28, 30, 32, 35 or 40°C) but a photoperiod of 22L: 2D was used in all of them. All larvae were fed ad libitum with horseradish leaves (Armoracia rusticana). Ovipositing adults were fed sugar water what was deployed on the white flowers of potted Kalanchoe sp. plants.
Wild animals	Wild-caught adult butterflies were transported in individual cups in a cooled and dark bag to the laboratory. Adult butterflies that survived until after all relevant data or eggs had been collected were killed through freezing (-80°C).
Reporting on sex	Butterfly individuals were sexed as pupae. Therefore, sex effects were estimated in life-stages where it was possible. For example, in larvae that survived until pupation, or in pupae, but not in eggs. In total, our experiments 1066 unsexed eggs, 381/240/270 unsexed/female/male larvae, 381/240/270 unsexed/female/male larvae, 25/240/264 unsexed/female/male pupae, and 419/146/157 unsexed/female/male individuals in the full ontogeny treatment.
Field-collected samples	No field collected samples were used.
Ethics oversight	This research project involved non-endangered invertebrates for which no ethical permit is required in Sweden.

Note that full information on the approval of the study protocol must also be provided in the manuscript.
